# Association of μ-Calpain and Calpastatin Polymorphisms with Meat Tenderness in a Brahman–Angus Population

**DOI:** 10.3389/fgene.2018.00056

**Published:** 2018-02-22

**Authors:** Joel D. Leal-Gutiérrez, Mauricio A. Elzo, Dwain D. Johnson, Tracy L. Scheffler, Jason M. Scheffler, Raluca G. Mateescu

**Affiliations:** Department of Animal Sciences, University of Florida, Gainesville, FL, United States

**Keywords:** bioinformatic analysis, calpain, calpastatin, putative functional SNPs, tenderness, transcription factor binding sites

## Abstract

Autogenous proteolytic enzymes of the calpain family are implicated in myofibrillar protein degradation. As a result, the μ-calpain gene and its specific inhibitor, calpastatin, have been repeatedly investigated for their association with meat quality traits in cattle; however, no functional mutation has been identified for these two genes. The objectives of this study were: (1) to assess breed composition effect on tenderness; (2) to perform a linkage disequilibrium (LD) analysis in μ-calpain and calpastatin genes as well as an association analyses with tenderness; and (3) to analyze putative functional SNPs inside the significant LD block for an effect on tenderness. Tenderness measurements and genotypes for 16 SNPs in μ-calpain gene and 28 SNPs in calpastatin gene from 673 steers were analyzed. A bioinformatic analysis identified “putative functional SNPs” inside the associated LD block – polymorphisms able to produce a physical and/or chemical change in the DNA, mRNA, or translated protein *in silico*. Breed composition had a significant (*P* < 0.0001) effect on tenderness where animals with more than 80% Angus composition had the most tender meat. One 11-kb LD-block and three LD-blocks of 37, 17, and 14 kb in length were identified in the μ-calpain and calpastatin genes, respectively. Out of these, the LD-block 3 in calpastatin, tagged by SNPs located at 7-98566391 and 7-98581038, had a significant effect on tenderness with the TG-CG diplotype being approximately 1 kg more tender than the toughest diplotype, TG-CG. A total of 768 SNPs in the LD-block 3 of calpastatin were included in the bioinformatic analysis, and 28 markers were selected as putative functional SNPs inside the LD-block 3 of calpastatin; however, none of them were polymorphic in this population. Out of 15 initial polymorphisms segregating inside the LD-block 3 of calpastatin in this population, markers ARSUSMARC116, Cast5, rs730723459, and rs210861835 were found to be significantly associated with tenderness.

## Introduction

Meat quality is determined by multiple factors, including tenderness, water-holding capacity, color, nutritional value and safety, and the importance of these traits varies depending on both the type of product and the consumer profile (Koohmaraie and Geesink, 2006). Tenderness has been established as the most important quality trait in beef, and it depends primarily on the amount of connective tissue, myofibrillar protein degradation (Houbak et al., 2008), and intramuscular fat content.

Myofibrillar protein degradation is a result of various autogenous proteolytic enzymes. Autogenous proteolytic enzymes of the calpain family are implicated in fundamental physiological processes, including cytoskeletal remodeling, cellular signaling, apoptosis, and cell survival (Storr et al., 2011). Three members of the calpain family of genes (μ-calpain, m-calpain, and calpain3) are well-established candidate genes because of their involvement in myofibrillar protein degradation. The μ-calpain gene was found to be responsible for postmortem muscle proteolysis in μ-calpain-knockout mice, even when m-calpain was active (Geesink et al., 2006). Similarly, postmortem proteolysis of muscle occurred in both control and calpain3-knockout mice (Geesink et al., 2005). Calpastatin, the specific inhibitor of calcium-dependent proteases, μ-calpain and m-calpain (Juszczuk-Kubiak et al., 2008) was shown to be involved in *in vivo* cell viability and cell proliferation (Van Ba et al., 2015). Further, over-expression of calpastatin significantly inhibited calpain activity in muscle, heart, and neuronal tissue (Li et al., 2009; Liang et al., 2010). Although many polymorphisms of μ-calpain and calpastatin were found associated with meat tenderness in different cattle populations (White et al., 2005; Casas et al., 2006; Morris et al., 2006; Barendse et al., 2007; Curi et al., 2009; Motter et al., 2009; Cafe et al., 2010; Chung and Davis, 2011; Tait et al., 2014), no functional mutations were identified. Thus, the objectives of this study were: (1) to assess breed composition effect on tenderness measured by Warner-Bratzler shear force (WBSF); (2) to perform a linkage disequilibrium (LD) analysis for μ-calpain and calpastatin genes as well as an association analyses with WBSF; and (3) to analyze putative functional SNPs inside the significant LD block for an effect on WBSF.

## Materials and Methods

### Cattle Population

The research protocol was approved by the University of Florida Institutional Animal Care and Use Committee number 201003744. Animals used in the study belong to the multibreed Angus–Brahman herd from University of Florida (Elzo et al., 2015a,b, 2016), born between 2007 and 2014. Cattle were classified into six different groups based on their expected Angus and Brahman breed composition. Based on the Angus composition, the grouping was as follows: 1 = 100 to 80%; 2 = 79 to 65%; 3 = 62.5% (Brangus); 4 = 59 to 40%; 5 = 39 to 20%; 6 = 19 to 0%.

When steers reached 1.27 cm subcutaneous fat thickness over the ribeye, they were transported to a commercial packing plant and harvested using established USDA-FSIS procedures. The average slaughter weight was 537.94 ± 55.31 kg at 17.31 ± 1.23 months. One 2.54 cm steak from the *Longissimus dorsi* muscle at the 12th/13th rib interface was sampled from each animal. Steaks were transported to the Meat Science Laboratory of the University of Florida, aged for 14 days at 1 to 4°C, and then stored at -20°C.

### Phenotypic Data

Tenderness was measured by WBSF on steaks from 673 steers according to the American Meat Science Association Sensory Guidelines (Belk et al., 2015). Frozen steaks were allowed to thaw at 4°C for 24 h, cooked to an internal temperature of 71°C on an open-hearth grill and cooled at 4°C for 18 to 24 h. From each steak, six cores with a 1.27-cm diameter and parallel to the muscle fiber were sheared with a Warner-Bratzler head attached to an Instron Universal Testing Machine (model 3343; Instron Corporation, Canton, MA, United States). The Warner-Bratzler head moved at a cross head speed of 200 mm/min. The average peak load (kg) of six cores from the same animal was calculated and was subsequently analyzed. The weight lost during cooking was recorded and cooking loss was expressed as percentage of the cooked weight out of the thaw weight.

### Genotyping

Genomic DNA was extracted from blood using the DNeasy Blood & Tissue kit (Qiagen, Valencia, CA, United States) and stored at -20°C. Genotypes for 15 SNPs in μ-calpain and 23 SNPs in calpastatin genes were obtained from the commercial SNP chip GGP Bovine F-250 (GeneSeek, Inc., Lincoln, NE, United States). One additional SNP in μ-calpain (Capn4751) and five SNPs in calpastatin (Cast1, Cast5, rs210861835, rs110747591, and rs384811952) were genotyped by high resolution melt (HRM) analysis on an Illumina’s Eco Real-Time PCR System (Illumina, United States). Primer sequences for the additional SNPs are presented in **Table 1**. Real-time PCR was performed in 10 μl volume reactions and included 10 ng of template genomic DNA, 5 μM of each primer (Integrated DNA Technologies, United States), 1X Fast EvaGreen (Biotium, United States), and sterile water. Amplification protocol started with 2 min at 50°C, 10 min at 95°C followed by 45 cycles of 95°C for 10 s, 62°C for 30 s, and 72°C for 15 s. Following the PCR amplification, the HRM analysis was performed by raising the temperature from 55 to 95°C at 0.1°C increments. Fluorescence was continuously monitored during the temperature increase. Specificity of each primer pair (absence of artifacts, multiple PCR products, or primer-dimers) and PCR yield was checked by agarose gel electrophoresis and melting analysis. One PCR product for each HRM-group was purified using a QIAquick PCR Purification Kit (Qiagen, Valencia, CA, United States) and sent for sequencing to confirm the corresponding genotype. The genotype data is available in the EVA website, accession number PRJEB24746.

**Table 1:** Primer sets for μ-calpain and calpastatin polymorphisms genotyped using high resolution melt analysis.

Primer set		Sequence	Size	Target polymorphism
Capn4751	F	5′-AAGAGCAGGGAAAGGGACAGAT-3′	134	Capn4751
	R	5′-CAGCCTTAGGGTCACCTGTAGA-3′		
Cast1	F	5′-CTTGCTGAATTTGGAGGGAAGG-3′	150	Cast1
	R	5′-TTTTCTCTGAGGAGTAGAAAGCAA-3′		
Cast5	F	5′-CTCACGTGTTCTTCAGTGTTCTG-3′	124	Cast5, rs210861835
	R	5′-ATGTGCCCAATGCACAGTATTTT-3′		rs110747591,rs384811952
CAST-Indel1-Block3	F	5′-TGGGCTTTTTCTTCTTGGCTTT-3′	150	rs730723459
	R	5′-CC-AAATAATGGTGTTTGGGGAGA-3′		rs380996268
CAST-Indel2-3-Block3	F	5′-TGACAAAAGCATATAACTACTCGC-3′	210	rs523462296, rs384162445
	R	5′-CTAAGGAGGCATTTACTTTAAGCAG-3′		rs380139611, rs378828670
CAST-Indel4-Block3	F	5′-TACCAGCCCGTGTGTTATAGGT-3′	133	rs382751934
	R	5′-AGCACCATTTATTGCTTTTCAGG-3′		
SNP1-CastBlock3	F	5′-TTTACAACAATACACTTGGGAATCA-3′	120	rs382321995
	R	5′-GGGTTCTTGGCCCGATTTCTA-3′		

*The sequence of the forward and reverse primer, the amplicon size (bp) and the target polymorphisms for each primer set are presented.*

### Statistical Analysis

Allelic and genotypic frequencies for each SNP were calculated using PROC FREQ procedure of SAS (SAS Institute, Inc., Cary, NC, United States). SNPs with minor allele frequencies (MAFs) lower than 10% and genotypic frequencies lower than 5% were excluded from the association analysis.

A total of 569 steers from 196 families were used for the LD-block construction using the Haploview software (Barrett et al., 2005). Any SNP with a MAF lower than 5% was excluded from this analysis, and a minimum 98% confidence interval for strong LD was used (Gabriel et al., 2002). Two or more tag SNPs were defined for each LD block.

The model used to analyze the effect of breed group on WBSF contained breed group and year of birth as fixed subclass effects, and cooking loss as a fixed covariate effect. Similarly, the model used to evaluate the association of SNP and haplotypes with WBSF included genotype, breed group and year of birth as fixed subclass effects, and cooking loss as a fixed covariate effect. Computations were carried out with the GLM procedure of SAS (SAS Institute, Inc., Cary, NC, United States).

A Bonferroni correction was used (α/n) to adjust for multiple SNP and haplotype tests. The Bonferroni significance level was 0.0029. Because of the known biological relationship between μ-calpain and calpastatin, interaction terms for pairs of SNPs were tested; however, none of them reached statistical significance.

#### Bioinformatic Analysis

A “putative functional SNP” was defined as a SNP able to produce a physical and/or chemical change in the DNA, mRNA or translated protein in an *in silico* analysis. To identify these polymorphisms, all reported SNPs in NCBI^1^ located inside the calpastatin LD-block associated with WBSF, or within 1 kb upstream or downstream of the LD block, were screened for putative function. The calpastatin gene sequence reported in NCBI (AC_000164.1) was considered the wild type (WT) sequence. Alternate sequences of the LD-block (alternate LD-block) were created by interchanging the reported alternative base at each SNP, for all the reported SNPs.

The source page for each reported SNP in the calpastatin LD-block was downloaded and an in-house Java script was used to create a unidimensional array with SNP name, flanking 3′ and 5′ sequences, reported base change, and chromosomal location. The *in silico* analysis included transcription factor binding sites (TFBSs), mRNA folding and stability, and isoelectric point and folding at the protein level.

The WT LD-block was analyzed with MATCHTM (Kel et al., 2003) to obtain the name and consensus recognition sequences for TFBS with expression only in skeletal muscle. Each one of the alternate LD-block sequences was analyzed with a Java in-house script to determine if the SNP added or deleted corresponded to any of the predicted TFBS.

The mRNA sequences of the WT and each alternate LD-block were analyzed with the RNAFold software (Zuker, 2003) for molecular stability (ΔG) and secondary structure.

To analyze changes in folding, surface structure and electrostatic potential or isoelectric point (IP) of the corresponding protein segment, SNPs located in the coding region were selected. A Java script translated the mRNA sequence of each alternate LD-block into protein sequence and compared them against the WT LD-block. Any sequence with missense SNPs was analyzed using SwissModel server (Biasini et al., 2014) and Compute pI tool (Bjellqvist et al., 1993) of ExPASy (Gasteiger et al., 2003; Arnold et al., 2006; Guex et al., 2009; Kiefer et al., 2009).

### Associated LD-Block Genotyping

To determine which of the putative functional SNPs identified through the bioinformatic analyses were polymorphic and segregating in our population, the calpastatin associated LD-block was subdivided into eight segments for sequencing. Five pools of DNA grouped according to the LD-block haplotype were sequenced for each of the eight segments. Out of 32 SNPs found in this population, four insertions/deletions (indels; rs730723459, rs523462296, rs380139611, and rs382751934) were selected for further genotyping by HRM. Six other SNPs (rs210861835, rs110747591, rs384811952, rs380996268, rs384162445, and rs378828670) located in close proximity to the four selected indels were also genotyped by HRM. All 15 genotyped markers in the associated LD-block were fit simultaneously in a regression model and a stepwise procedure was performed to establish the most significant markers.

## Results

### Warner-Bratzler Shear Force

Tenderness measured by WBSF ranged from 1.5 to 7.4 kg. The effect of breed composition is shown in **Table 2**. A significant breed group effect on WBSF (*P* < 0.0001) was identified, with animals with more than 80% Angus composition having the most tender meat.

**Table 2:** Effect of breed composition on meat tenderness measured by Warner-Bratzler shear force (WBSF).

Breed composition	*n*	WBSF (kg)	*SE*
100–80% Angus	112	3.91^a^	0.01
79–65% Angus	97	4.11^ab^	0.01
62.5% Angus	113	4.07^ab^	0.01
59–40% Angus	144	4.20^b^	0.01
39–20% Angus	66	4.46^c^	0.02
19–0% Angus	146	4.45^c^	0.01

*Breed group based on Angus percentage, number of animals (n), least square means (LSM), and SE for WBSF in *Longissimus dorsi* muscle in an Angus–Brahman crossbred population. LSM without a common superscript are significantly different (*P* < 0.05).*

### Gene and Genotypic Frequencies

**FIGURE 1:**
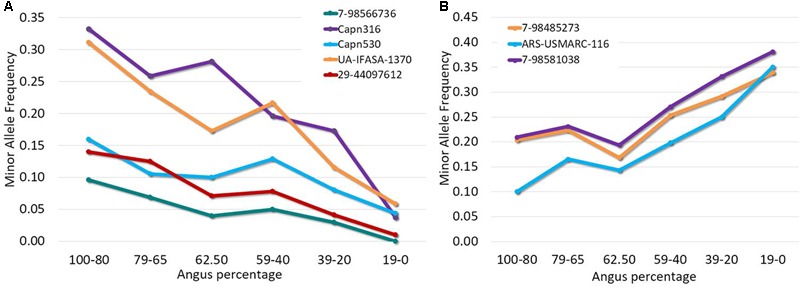
Minor allele frequency for selected μ-calpain and calpastatin SNPs across six breed groups present in an Angus-Brahman crossbred population. **(A)** Contains SNPs whose MAF decreases when Angus percentage decreases; **(B)** includes SNPs whose MAF increases when Angus percentage decreases.

Out of a total of 44 SNPs in μ-calpain and calpastatin genes, 28 SNPs were segregating in this population. Allele and genotypic frequencies are shown in the Supplementary Table 1. Allelic frequencies for several SNPs varied substantially across breed groups of different breed composition, with MAF either decreasing or increasing as Angus percentage increased (**Figure 1**). The biggest difference in allele frequency across breed groups was observed for Capn316, UA-IFASA-1370, and 7-98581038.

### Predicted LD Blocks and Haplotypes

**FIGURE 2:**
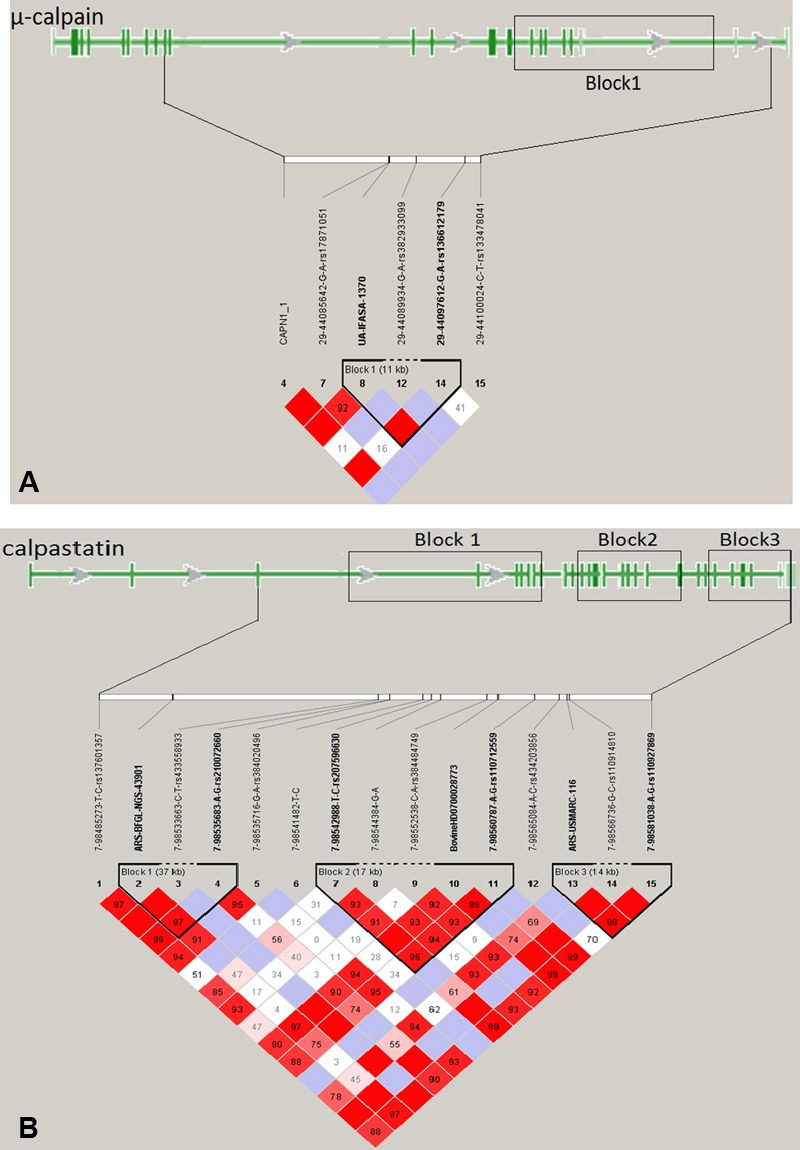
Predicted linkage disequilibrium blocks for 569 steers from 196 families in an Angus-Brahman crossbred population. **(A)** μ-calpain gene; **(B)** calpastatin gene.

One 11-kb LD-block was identified in the μ-calpain gene (**Figure 2A**), with two intronic SNPs (UA-IFASA-1370 and 29-44097612) as corresponding tag SNPs. Based on the NCBI isoform NM_174259.2, the LD-block includes seven exons (from exon 15 to exon 22) with the last one being part of the 3′ UTR. Three LD-blocks of 37, 17, and 14 kb in length, respectively, were identified for calpastatin (**Figure 2B**). Based on the NCBI isoform XM_005209749.2, calpastatin LD-block 1 is located between the tag SNPs ARS-BFGL-NGS-43901 (intron 3) and 7-98535683 (exon 9). LD-block 2 has three tag SNPs: 7-98542988 (intron 12), BovineHD0700028773 (exon 20), and 7-98560787 (exon 22) and covers 10 exons. The tag SNPs of the LD-block 3 are ARS-USMARC-116 (intron 25) and 7-98581038 (exon 31). This 14 kb block is located at the terminal end of calpastatin and includes a segment of the 3′ UTR (**Figure 2B**).

### SNP and Haplotype Association Analysis

Out of 12 SNPs from the 250K bovine SNP BeadChip and three SNPs genotyped by HRM, only ARSUSMARC116 (7-98566391) showed a significant association (*P* = 0.0006) with WBSF (**Table 3**). The CC genotype at this locus was associated with tougher meat (4.73 ± 0.10 kg) and was significantly different from both the TT (4.14 ± 0.17 kg) and CT (4.28 ± 0.11 kg) genotypes. This SNP is also the tag SNP for the calpastatin LD-block 3, which was also associated with WBSF. Using genotypes of the tag SNPs ARSUSMARC116 and 7-98581038, five distinct diplotypes were constructed for the calpastatin LD-block 3. The TG-CG diplotype was associated with the most tender phenotype with a WBSF of 3.77 ± 0.24 kg, which was approximately 1 kg more tender than the toughest diplotype, TG-CG.

**Table 3:** Significant effects of one SNP and one LD-block in calpastatin gene on WBSF of *Longissimus dorsi* muscle in an Angus–Brahman crossbred population.

	Genotype/diplotype	*n*	WBSF (kg)	*SE*	*P-*Value
ARSUSMARC116	TT	436	4.14^b^	0.17	0.0006
	CT	196	4.28^b^	0.11	
	CC	41	4.73^a^	0.1	
LD-block 3	TG-CG	16	3.77^a^	0.24	0.0003
	TA-TA	356	4.13^a^	0.11	
	TA-TG	66	4.15^ab^	0.13	
	TA-CG	178	4.32^b^	0.12	
	CG-CG	39	4.75^c^	0.18	

*Diplotype of the LD-block 3 was based on the tag SNPs ARSUSMARC116 and 7-98581038, with the first and third position representing the two ARSUSMARC116 alleles. Least square means for WBSF without a common superscript are significantly different.*

### Bioinformatic Analysis

A total number of 768 SNPs in the LD-block 3 of calpastatin were included in the TFBS bioinformatics analysis, 52 SNPs were located in exonic regions including the 3′ UTR and were considered in the mRNA analysis, and 45 SNPs in translated exonic regions were analyzed at the protein level. A total number of 28 SNPs were identified through these analyses as putative functional SNPs. Twelve SNPs were selected from the TFBS analysis with one SNP creating a new TFBS compared to the WT sequence and 11 SNPs eliminating at least one TFBS. Seven SNPs were selected from the mRNA analysis based on their ability to alter mRNA folding or stability. From the protein test, 13 SNPs modify the isoelectric point and four SNPs modify protein surface and folding. The selected 28 putative functional SNPs were distributed in eight different regions of the LD-block 3 of calpastatin (Supplementary Table 2).

### Analysis of Putative Functional SNPs

None of the initial 28 putative functional SNPs identified through bioinformatics analyses were polymorphic. During sequencing of the calpastatin LD-block 3, 32 polymorphisms including four indels that were segregating in this population were identified (Supplementary Table 2).

### Regression Model Selection

Out of 15 initial polymorphisms (Supplementary Table 1) found in this population inside the LD-block 3 of calpastatin, four were shown to be significantly associated with WBSF through a stepwise regression procedure: ARSUSMARC116 (*P-*value = 0.0099), Cast5 (*P-*value = 0.0080), rs730723459 (*P-*value = 0.0014), and rs210861835 (*P-*value = 0.0007). The favorable alleles were T in ARSUSMARC116, T in Cast5, insertion AC in rs730723459 and T in rs210861835.

## Discussion

The main effect of μ-calpain and calpastatin on WBSF is through muscle proteolysis. To account for effects of cooking loss on tenderness unrelated to muscle proteolysis (Kołczak et al., 2007; Bouhrara et al., 2011, 2012), cooking loss was included as a covariate in the association analysis. Leal-Gutiérrez et al. (2015) found a highly significant association between the Cast5 marker and water holding capacity in raw meat. However, Cast5 had no significant effect on cooking loss.

### The μ-Calpain Gene

Several SNPs in μ-calpain have been repeatedly studied for their association with meat tenderness including Capn316, Capn530, and Capn4751. All three SNPs were genotyped and found to be polymorphic in this population; however, no significant association with meat tenderness was identified. Breed composition had an effect on MAF of Capn316 and Capn530, with MAF increasing gradually as Angus percentage increased (**Figure 1**). A similar effect was reported previously, more noticeably in *Bos indicus* breeds (Morris et al., 2006; Parra et al., 2015). Pinto et al. (2010) found a low MAF for Capn316 in a Nellore population and Papaleo et al. (2010) reported similar findings in a Brangus population. Capn316 has been shown to be associated with tenderness in multiple populations. Pinto et al. (2010) reported the C allele to be associated with increased tenderness in Nellore cattle, with the contrast CG–GG having values of -0.71, -1.1, and -0.69 kg of WBSF at 7, 14 and 21 days postmortem, respectively. White et al. (2005) assessed four independent populations including *Bos indicus, Bos taurus*, and crossbreds. A significant association between Capn316 and tenderness was found in 597 steers from Beefmaster, Brangus, Bonmara, Romosinuano, and Hereford–Angus sires mated to Angus or multibreed cows. White et al. (2005) determined that the favorable allele was C, and the CC genotype was approximately 0.55 kg more tender than the GG genotype. Casas et al. (2005) reported the G allele to have a favorable effect, with the CG genotype averaging a tenderness score of 5.34 ± 0.20 compared to 4.89 ± 0.04 for the GG genotype in a population of *Bos indicus* cattle. Conversely, White et al. (2005) found no association between Capn316 and tenderness in a purebred Brahman population (*n* = 504).

In the present study, the frequency of C allele (minor allele) of Capn530 was 16% in the group with the highest Angus composition (80 to 100% Angus) and decreased as the Brahman percentage increased, reaching its lowest frequency (5%) in the 80 to 100% Brahman breed group. White et al. (2005) found a similar trend for this marker with a poor segregation in the populations under investigation. In a different Brahman population, the Capn530 marker was not informative (Casas et al., 2005). Similarly, Costello et al. (2007) found no association between a Capn530 polymorphism and WBSF in *longissimus dorsi* at 14 days postmortem. Corva et al. (2007) found that meat from pure and crossbred Limousin, Angus and Hereford steers with genotype GG (8.90 ± 0.47 kg) had higher WBSF values than GA animals (7.98 ± 0.62 kg). The C allele had a frequency of 39% across all breed groups in the population analyzed by Corva et al. (2007).

White et al. (2005) affirmed that Capn4751 might be the only useful marker in populations with a high percentage of *Bos indicus* influence where the favorable C allele of Capn316 is rare. However, this marker was not associated with WBSF in the present population, highlighting the importance of validating any marker identified in other studies in the target population. Pinto et al. (2010) reported that the favorable allele of Capn4751 was C and the difference between animals with CC versus TT genotypes was estimated to be -0.30, -0.27, and -0.34 kg of shear force at 7, 14, and 21 days postmortem, respectively. Association between Capn4751 and WBSF or tenderness was also reported by Casas et al. (2006), White et al. (2005), Curi et al. (2009), Cafe et al. (2010), and McClure et al. (2012).

### Calpastatin Gene

The ARSUSMARC116 was associated with WBSF in the present population and this association was significant when the SNP was analyzed individually or as a tag SNP for the calpastatin LD-block 3. When considering the entire calpastatin LD-block 3, the diplotypes constructed based on the tag SNPs were able to partition the population for WBSF with higher specificity. The two extreme diplotypes had a WBSF of 4.75 kg (CG-CG diplotype) and 3.77 kg (TG-CG diplotype). Breed group affected the allelic frequency of both tag SNPs for the LD-block 3 (ARSUSMARC116 and 7-98581038) as it was previously described for the SNPs of the μ-calpain gene. The frequency of the minor allele (allele C for ARSUSMARC116 and allele G for 7-98581038) decreased from 35 and 37% in the highest percentage Brahman breed group to 10 and 21% in the breed group with the highest Angus composition, respectively. Because of the similar trend in frequencies for tag SNPs, diplotype frequencies showed the same trend, indicating that segments nearby and between ARSUSMARC116-C and 7-98581038-G had higher frequency in purebred and crossed Brahman subgroups in this population.

It is somewhat surprising that, in spite of a multitude of putative functional SNPs identified through the bioinformatic analysis, none of them were segregating in the present population. As indicated above, calpastatin is a perfect candidate gene based on its biological function and its association with beef tenderness in prior studies. The Angus–Brahman crossbred population used for the current study was also expected to maximize the segregation of functional polymorphisms related to tenderness. Angus cattle have been selected for increased meat quality and have more tender meat compared to *Bos indicus* cattle, therefore the crossbreds are expected to segregate a higher percentage of tenderness related alleles, which may be fixed in the two purebred populations. Therefore, our sequencing efforts were concentrated on the region that showed an association with WBSF in this population, although it is possible that the functional mutation is located in a different region of the gene. Recent reports highlight the possibility of multiple polymorphisms, each one with a small effect, being responsible for phenotypic variation previously assigned to a locus. Bickel et al. (2011) assessed a locus that contributes to abdominal pigmentation in *Drosophila melanogaster* and found that the large phenotypic effect of this locus resulted from the cumulative action of many small-effect polymorphisms that were concentrated in three distinct functional regions of this locus.

### Significant Polymorphisms in the Calpastatin LD-Block 3

#### The cast5 and rs210861835 Polymorphisms

Stability of mRNA and conformational polymorphism has been reported as possible regulation mechanisms of gene expression. Analyses of SNPs within known functional RNA structures (iron response elements, selenocysteine insertion sequences, micro RNAs, and small nucleolar RNAs) identified a number of putative functional SNPs (Jonhson et al., 2011), many of them within genes known to be associated with certain phenotypes, suggesting that conformational RNA polymorphisms substantially contribute to phenotypic variability (Jonhson et al., 2011). The RNA contains both analogous and digital information. Digital information is determined by the “text” that can be read linearly from the mRNA, and analogous information is based on the tridimensional structure of these molecules (Andrade, 2011). This tridimensional structure allows for substrate recognition and consequently molecular activity. Thus, polymorphisms in a transcribed region of a gene can alter mRNA structure, cellular half-life, and ribosome processivity/elongation (Hamasaki et al., 2017).

The Cast5 (rs109221039) T and rs210861835 C alleles were predicted to have increased molecular stability (lower ΔG) and theoretically produce a more stable mRNA, which would result in more calpastatin protein and subsequently a higher μ-calpain inhibition rate. However, only rs210861835 C allele was determined to have an unfavorable effect on WBSF in the present study, while the Cast5 T allele had a favorable effect.

The importance of the UTR regions for the stability and folding of the mRNAs has been previously demonstrated, and polymorphisms in the UTR regions tend to have a more pronounced deleterious effect compared to polymorphisms in coding regions (Jonhson et al., 2011). Halvorsen et al. (2010) reported that the majority of SNPs associated with Hyperferritinemia Cataract Syndrome occur in the 5′ UTR of the human ferritin light chain (*FTL*) gene; however, the majority of mutations result only in changes in mRNA structure. Duan et al. (2003) investigated *in vitro* the translation of the human dopamine receptor D2 (*DRD2*) mRNAs carrying various synonymous mutations. When the protein synthesis was compared, it was found that *DRD2* mRNA carrying the 957T mutation showed a 50% decrease in protein synthesis. Therefore, differences in protein translation efficiency could result from differences in structure of the corresponding mRNAs (Nackley et al., 2006) and/or from changes in mRNA stability.

**FIGURE 3:**
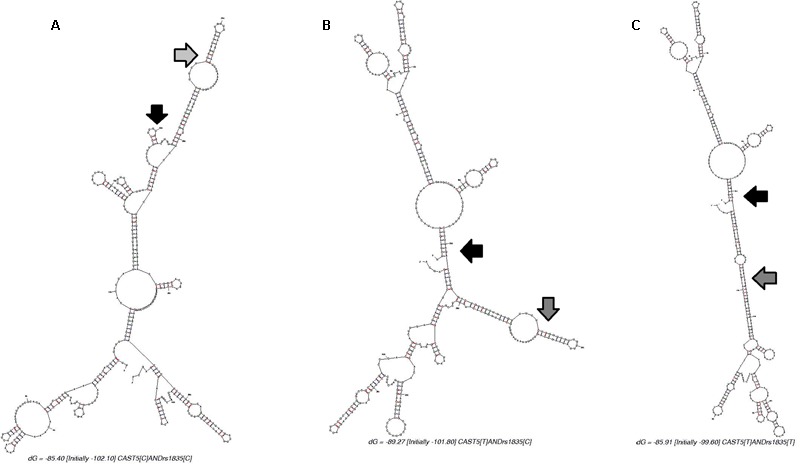
Predicted mRNA structure of a 429 bp segment of the calpastatin gene harboring the Cast5 and rs210861835 polymorphisms (200 bp upstream of Cast5 and 200 bp downstream of rs210861835). The black and gray arrows indicate the location of Cast5 and rs210861835, respectively. **(A)**. Folding for Cast5-C/rs210861835-C (ΔG = -85.40 kcal/mol) or Cast5-C/rs210861835-T (ΔG = -83.20 kcal/mol). **(B)**. Folding for Cast5-T/rs210861835-C (ΔG = -89.27 kcal/mol). **(C)**. Folding for Cast5-T/rs210861835-T (ΔG = -85.91 kcal/mol).

Regarding Cast5, it is possible that changes in mRNA structure allow for higher protein expression even when the mRNA molecule is theoretically more unstable. The predicted molecular folding of an mRNA segment harboring the Cast5 and rs210861835 alleles is shown in **Figure 3**. The Cast5 polymorphism seems to have a larger effect on mRNA structure than rs210861835. Bartoszewski et al. (2010) demonstrated that the ΔF508 mutation alters the mRNA structure of the human cystic fibrosis transmembrane conductance regulator (*CFTR*) gene. The “misfolded” *CFTR* mRNA decreased translational rate. However, this misfolding of the mutant mRNA does not decrease mRNA stability, but there are enlarged single-stranded regions in the vicinity of the mutation and these regions slow down translation (Bartoszewski et al., 2010). This change in translation speed could explain the Cast5 effect on WBSF. The C to T change minimizes the single-stranded regions (**Figures 3B,C**). **Figure 3A** shows a higher number, frequency, and extension of these regions. Another possible mechanism of regulation of mRNA liability and final amount of calpastatin protein could be related to changes in folding of the UTR region, resulting in changes in miRNA-mediated regulation and degradation or a direct change in miRNA binding sites in calpastatin (Haas et al., 2012).

This association between Cast5 and meat quality traits in beef has been reported previously. Casas et al. (2006) found an association with WBSF, tenderness score and juiciness in the *longissimus thoracis* muscle aged 14 days postmortem. Animals inheriting the CC and the CT genotypes produced tougher meat, measured by WBSF and sensory panel, when compared with animals that inherited the TT genotype. Li et al. (2010) found an association between WBSF and Cast5 in 212 cattle from diverse genetic structure. They showed that animals with the CC genotype (5.13 ± 0.28 kg) had significantly higher WBSF than those with CT (4.01 ± 0.22 kg) or TT (3.98 ± 0.19 kg) genotypes. Curi et al. (2009) showed that Cast5 was significantly associated with shear force (SF) and the myofibrillar fragmentation index, reporting the T allele as the favorable one. An association between Cast5 and water holding capacity in raw meat in a crossbred *Bos indicus–Bos taurus* population was found by Leal-Gutiérrez et al. (2015). McClure et al. (2012) assessed the association of 28 SNPs in calpastatin with WBSF of *Longissimus dorsi* steaks of 3360 animals. This population was represented by purebred Angus and Hereford animals and crossbred progeny of Simmental, Charolais and Limousin sires mated to Angus dams. One SNP located 89 bp up-stream of Cast5, rs41255587, had the strongest association with WBSF across-breed and within Charolais and Hereford; some other SNPs in the 5′ upstream region of calpastatin had strong association within Angus, Limousin and Simmental. In the same study, a sliding window analysis identified a calpastatin window approximately 7.5 kb up-stream of ARS-USMARC-116 able to explain the greatest amount of phenotypic variation in WBSF across breeds.

#### The rs730723459 Polymorphism

The rs730723459 indel belongs to a dinucleotide short tandem repeat (STR), and it is located 382 bp upstream from the TFSB for c-Rel (recognition sequence TGGGCTTTCC). It has been shown that variation in the length of an STR can affect gene regulation (Sarrió et al., 2003; Giannini et al., 2004; Yim et al., 2007; Sawaya et al., 2013). Meloni et al. (1998) determined that an STR located in the first intron of the tyrosine hydroxylase gene is associated with genetic predisposition to schizophrenia. While both alleles of this STR act as enhancers of transcription, there was a different efficacy in nuclear factor binding. This showed that STRs could regulate gene transcription by changing the interaction between transcription factors and the regulated gene.

The c-Rel transcription factor is part of the homo- or heterodimeric NF-kappa-B complex which can act as transcriptional activator or repressor of transcriptional activity. NF-kappa-B is a pleiotropic TF present in almost all cell types and involved in many biological processes such as inflammation, immunity, differentiation, cell growth, tumorigenesis, and apoptosis^2^.

## Conclusion

In the present population, four markers in the calpastatin gene were significantly associated with WBSF: ARSUSMARC116 (*P* = 0.0099), Cast5 (*P* = 0.0080), rs730723459 (*P* = 0.0014), and rs210861835 (*P* = 0.0007). The favorable alleles were T in ARSUSMARC116, T in Cast5, insertion AC in rs730723459 and T in rs210861835. The rs210861835 and Cast5 SNPs are predicted to regulate gene expression through mRNA stability and mRNA conformational polymorphism. The rs210861835 C allele was predicted to have an increased molecular stability (lower ΔG) and theoretically a more lasting mRNA, which would result in more calpastatin protein and subsequently a higher μ-calpain inhibition rate. Cast5 changes the structure of mRNA and increases the number and length of single-stranded regions in the vicinity of this polymorphism leading to higher protein expression. Finally, the rs730723459 indel belongs to a dinucleotide STR located 16 bp downstream from the TFBS for BR-C Z1 and 382 bp upstream from the TFSB for c-Rel.

## Author Contributions

JL-G conducted all analyses and drafted the manuscript. ME assisted with the analysis and manuscript. DJ, TS, and JS assisted with data collection and manuscript. RM conceived and assisted with the analyses and manuscript.

## Conflict of Interest Statement

The authors declare that the research was conducted in the absence of any commercial or financial relationships that could be construed as a potential conflict of interest.

## Footnotes

1 www.ncbi.nlm.nih.gov

2 www.genecards.org
